# A manifesto for reproducible science

**DOI:** 10.1038/s41562-016-0021

**Published:** 2017-01-10

**Authors:** Marcus R. Munafò, Brian A. Nosek, Dorothy V. M. Bishop, Katherine S. Button, Christopher D. Chambers, Nathalie Percie du Sert, Uri Simonsohn, Eric-Jan Wagenmakers, Jennifer J. Ware, John P. A. Ioannidis

**Affiliations:** 1 MRC Integrative Epidemiology Unit, University of Bristol, Bristol BS8 2BN, UK; 2 UK Centre for Tobacco and Alcohol Studies, School of Experimental Psychology, University of Bristol, 12a Priory Road, Bristol BS8 1TU, UK; 3 Department of Psychology, University of Virginia, Charlottesville, Virginia 22904, USA; 4 Center for Open Science, Charlottesville, Virginia 22903, USA; 5 Department of Experimental Psychology, University of Oxford, 9 South Parks Road, Oxford OX1 3UD, UK; 6 Department of Psychology, University of Bath, Bath BS2 7AY, UK; 7 Cardiff University Brain Research Imaging Centre, School of Psychology, Cardiff University, Cardiff CF24 4HQ, UK; 8 National Centre for the Replacement, Refinement and Reduction of Animals in Research (NC3Rs), London NW1 2BE, UK; 9 The Wharton School, University of Pennsylvania, Philadelphia, Pennsylvania 19104, USA; 10 Department of Psychology, University of Amsterdam, Amsterdam 1018 WT, Netherlands; 11 CHDI Management/CHDI Foundation, New York, New York 10001, USA; 12 Meta-Research Innovation Center at Stanford (METRICS), Stanford University, Stanford 94304, California, USA; 13 Stanford Prevention Research Center, Department of Medicine and Department of Health Research and Policy, Stanford University School of Medicine, Stanford 94305, California, USA; 14 Department of Statistics, Stanford University School of Humanities and Sciences, Stanford 94305, California, USA

## Abstract

Improving the reliability and efficiency of scientific research will increase the credibility of the published scientific literature and accelerate discovery. Here we argue for the adoption of measures to optimize key elements of the scientific process: methods, reporting and dissemination, reproducibility, evaluation and incentives. There is some evidence from both simulations and empirical studies supporting the likely effectiveness of these measures, but their broad adoption by researchers, institutions, funders and journals will require iterative evaluation and improvement. We discuss the goals of these measures, and how they can be implemented, in the hope that this will facilitate action toward improving the transparency, reproducibility and efficiency of scientific research.

What proportion of published research is likely to be false? Low sample size, small effect sizes, data dredging (also known as *P*-hacking), conflicts of interest, large numbers of scientists working competitively in silos without combining their efforts, and so on, may conspire to dramatically increase the probability that a published finding is incorrect^1^. The field of metascience — the scientific study of science itself — is flourishing and has generated substantial empirical evidence for the existence and prevalence of threats to efficiency in knowledge accumulation (refs 2–7; Fig. 1).

Data from many fields suggests reproducibility is lower than is desirable^8–14^; one analysis estimates that 85% of biomedical research efforts are wasted^14^, while 90% of respondents to a recent survey in *Nature* agreed that there is a ‘reproducibility crisis’^15^. Whether ‘crisis’ is the appropriate term to describe the current state or trajectory of science is debatable, but accumulated evidence indicates that there is substantial room for improvement with regard to research practices to maximize the efficiency of the research community’s use of the public’s financial investment in research.

Here we propose a series of measures that we believe will improve research efficiency and robustness of scientific findings by directly targeting specific threats to reproducible science. We argue for the adoption, evaluation and ongoing improvement of these measures to optimize the pace and efficiency of knowledge accumulation. The measures are organized into the following categories^16^: methods, reporting and dissemination, reproducibility, evaluation and incentives. They are not intended to be exhaustive, but provide a broad, practical and evidence-based set of actions that can be implemented by researchers, institutions, journals and funders. The measures and their current implementation are summarized in Table 1.

## The problem

A hallmark of scientific creativity is the ability to see novel and unexpected patterns in data. John Snow’s identification of links between cholera and water supply^17^, Paul Broca’s work on language lateralization^18^ and Jocelyn Bell Burnell’s discovery of pulsars^19^ are examples of breakthroughs achieved by interpreting observations in a new way. However, a major challenge for scientists is to be open to new and important insights while simultaneously avoiding being misled by our tendency to see structure in randomness. The combination of apophenia (the tendency to see patterns in random data), confirmation bias (the tendency to focus on evidence that is in line with our expectations or favoured explanation) and hindsight bias (the tendency to see an event as having been predictable only after it has occurred) can easily lead us to false conclusions^20^. Thomas Levenson documents the example of astronomers who became convinced they had seen the fictitious planet Vulcan because their contemporary theories predicted its existence^21^. Experimenter effects are an example of this kind of bias^22^.

Over-interpretation of noise is facilitated by the extent to which data analysis is rapid, flexible and automated^23^. In a high-dimensional dataset, there may be hundreds or thousands of reasonable alternative approaches to analysing the same data^24,25^. For example, in a systematic review of functional magnetic resonance imaging (fMRI) studies, Carp showed that there were almost as many unique analytical pipelines as there were studies^26^. If several thousand potential analytical pipelines can be applied to high-dimensional data, the generation of false-positive findings is highly likely. For example, applying almost 7,000 analytical pipelines to a single fMRI dataset resulted in over 90% of brain voxels showing significant activation in at least one analysis^27^.

During data analysis it can be difficult for researchers to recognize *P*-hacking^28^ or data dredging because confirmation and hindsight biases can encourage the acceptance of outcomes that fit expectations or desires as appropriate, and the rejection of outcomes that do not as the result of suboptimal designs or analyses. Hypotheses may emerge that fit the data and are then reported without indication or recognition of their *post hoc* origin^7^. This, unfortunately, is not scientific discovery, but self-deception^29^. Uncontrolled, it can dramatically increase the false discovery rate. We need measures to counter the natural tendency of enthusiastic scientists who are motivated by discovery to see patterns in noise.

## Methods

In this section we describe measures that can be implemented when performing research (including, for example, study design, methods, statistics, and collaboration).

### Protecting against cognitive biases

There is a substantial literature on the difficulty of avoiding cognitive biases. An effective solution to mitigate self-deception and unwanted biases is blinding. In some research contexts, participants and data collectors can be blinded to the experimental condition that participants are assigned to, and to the research hypotheses, while the data analyst can be blinded to key parts of the data. For example, during data preparation and cleaning, the identity of experimental conditions or the variable labels can be masked so that the output is not interpretable in terms of the research hypothesis. In some physical sciences this approach has been extended to include deliberate perturbations in or masking of data to allow data preparation (for example, identification of outliers) to proceed without the analyst being able to see the corresponding results^30^. Pre-registration of the study design, primary outcome(s) and analysis plan (see ‘Promoting study pre-registration’ section’ below) is a highly effective form of blinding because the data do not exist and the outcomes are not yet known.

### Improving methodological training

Research design and statistical analysis are mutually dependent. Common misperceptions, such as the interpretation of *P* values^31^, limitations of null-hypothesis significance testing^32^, the meaning and importance of statistical power^2^, the accuracy of reported effect sizes^33^, and the likelihood that a sample size that generated a statistically significant finding will also be adequate to replicate a true finding^34^, could all be addressed through improved statistical training. Similarly, basic design principles are important, such as blinding to reduce experimenter bias, randomization or counterbalancing to control for confounding, and the use of within-subjects designs, where possible, to maximize power. However, integrative training in research practices that can protect oneself against cognitive biases and the effects of distorted incentives is arguably more important. Moreover, statistical and methodological best practices are under constant revision and improvement, so that senior as well as junior researchers need continuing methodological education, not least because much training of early-career researchers is informal and flows from their supervisor or mentor. A failure to adopt advances in methodology — such as the very slow progress in increasing statistical power^35,36^ — may be partly a function of failing to inculcate a continuing professional education and development ethic.

Without formal requirements for continuing education, the most effective solutions may be to develop educational resources that are accessible, easy-to-digest and immediately and effectively applicable to research (for example, brief, web-based modules for specific topics, and combinations of modules that are customized for particular research applications). A modular approach simplifies the process of iterative updating of those materials. Demonstration software and hands-on examples may also make the lessons and implications particularly tangible to researchers at any career stage: the Experimental Design Assistant (https://eda.nc3rs.org.uk) supports research design for whole animal experiments, while *P*-hacker (http://shinyapps.org/apps/p-hacker/) shows just how easy it is to generate apparently statistically significant findings by exploiting analytic flexibility.

### Implementing independent methodological support

The need for independent methodological support is well-established in some areas — many clinical trials, for example, have multidisciplinary trial steering committees to provide advice and oversee the design and conduct of the trial. The need for these committees grew out of the well-understood financial conflicts of interest that exist in many clinical trials. The sponsor of a trial may be the company manufacturing the product, and any intentional or unintentional influence can distort the study design, analysis and interpretation of results for the ultimate financial benefit of the manufacturer at the cost of the accuracy of the science and the health benefit to the consumers^37,38^. Non-financial conflicts of interest also exist, such as the beliefs and preconceptions of individual scientists and the stakes that researchers have in obtaining publishable results in order to progress their career^39,40^. Including independent researchers (particularly methodologists with no personal investment in a research topic) in the design, monitoring, analysis or interpretation of research outcomes may mitigate some of those influences, and can be done either at the level of the individual research project or through a process facilitated by a funding agency (see Box 1).

### Encouraging collaboration and team science

Studies of statistical power persistently find it to be below (sometimes well below) 50%, across both time and the different disciplines studied^2,35,36^. Low statistical power increases the likelihood of obtaining both false-positive and false-negative results^2^, meaning that it offers no advantage if the purpose is to accumulate knowledge. Despite this, low-powered research persists because of dysfunctional incentives, poor understanding of the consequences of low power, and lack of resources to improve power. Team science is a solution to the latter problem — instead of relying on the limited resources of single investigators, distributed collaboration across many study sites facilitates high-powered designs and greater potential for testing generalizability across the settings and populations sampled. This also brings greater scope for multiple theoretical and disciplinary perspectives, and a diverse range of research cultures and experiences, to be incorporated into a research project.

Multi-centre and collaborative efforts have a long and successful tradition in fields such as randomized controlled trials in some areas of clinical medicine, and in genetic association analyses, and have improved the robustness of the resulting research literatures. Multi-site collaborative projects have also been advocated for other types of research, such as animal studies^41–43^, in an effort to maximize their power, enhance standardization, and optimize transparency and protection from biases. The Many Labs projects illustrate this potential in the social and behavioural sciences, with dozens of laboratories implementing the same research protocol to obtain highly precise estimates of effect sizes, and evaluate variability across samples and settings^44,45^. It is also possible, and desirable, to incorporate a team science ethos into student training (see Box 2).

## Reporting and dissemination

In this section we describe measures that can be implemented when communicating research (including, for example, reporting standards, study pre-registration, and disclosing conflicts of interest).

### Promoting study pre-registration

Pre-registration of study protocols for randomized controlled trials in clinical medicine has become standard practice^46^. In its simplest form it may simply comprise the registration of the basic study design, but it can also include a detailed pre-specification of the study procedures, outcomes and statistical analysis plan. It was introduced to address two problems: publication bias and analytical flexibility (in particular outcome switching in the case of clinical medicine). Publication bias^47^, also known as the file drawer problem^48^, refers to the fact that many more studies are conducted than published. Studies that obtain positive and novel results are more likely to be published than studies that obtain negative results or report replications of prior results^47,49,50^. The consequence is that the published literature indicates stronger evidence for findings than exists in reality. Outcome switching refers to the possibility of changing the outcomes of interest in the study depending on the observed results. A researcher may include ten variables that could be considered outcomes of the research, and — once the results are known — intentionally or unintentionally select the subset of outcomes that show statistically significant results as the outcomes of interest. The consequence is an increase in the likelihood that reported results are spurious by leveraging chance, while negative evidence gets ignored. This is one of several related research practices that can inflate spurious findings when analysis decisions are made with knowledge of the observed data, such as selection of models, exclusion rules and covariates. Such data-contingent analysis decisions constitute what has become known as *P*-hacking^51^, and pre-registration can protect against all of these.

The strongest form of pre-registration involves both registering the study (with a commitment to make the results public) and closely pre-specifying the study design, primary outcome and analysis plan in advance of conducting the study or knowing the outcomes of the research. In principle, this addresses publication bias by making all research discoverable, whether or not it is ultimately published, allowing all of the evidence about a finding to be obtained and evaluated. It also addresses outcome switching, and *P*-hacking more generally, by requiring the researcher to articulate analytical decisions prior to observing the data, so that these decisions remain data-independent. Critically, it also makes clear the distinction between data-independent confirmatory research that is important for testing hypotheses, and data-contingent exploratory research that is important for generating hypotheses.

While pre-registration is now common in some areas of clinical medicine (due to requirements by journals and regulatory bodies, such as the Food and Drug Administration in the United States and the European Medicines Agency in the European Union), it is rare in the social and behavioural sciences. However, support for study pre-registration is increasing; websites such as the Open Science Framework (http://osf.io/) and AsPredicted (http://AsPredicted.org/) offer services to pre-register studies, the Preregistration Challenge offers education and incentives to conduct pre-registered research (http://cos.io/prereg), and journals are adopting the Registered Reports publishing format^52,53^ to encourage pre-registration and add results-blind peer review (see Box 3).

### Improving the quality of reporting

Pre-registration will improve discoverability of research, but discoverability does not guarantee usability. Poor usability reflects difficulty in evaluating what was done, in reusing the methodology to assess reproducibility, and in incorporating the evidence into systematic reviews and metaanalyses. Improving the quality and transparency in the reporting of research is necessary to address this. The Transparency and Openness Promotion (TOP) guidelines offer standards as a basis for journals and funders to incentivize or require greater transparency in planning and reporting of research^54^. TOP provides principles for how transparency and usability can be increased, while other guidelines provide concrete steps for how to maximize the quality of reporting in particular areas. For example, the Consolidated Standards of Reporting Trials (CONSORT) statement provides guidance for clear, complete and accurate reporting of randomized controlled trials^55–57^. Over 300 reporting guidelines now exist for observational studies, prognostic studies, predictive models, diagnostic tests, systematic reviews and meta-analyses in humans, a large variety of studies using different laboratory methods, and animal studies. The Equator Network (http://www.equator-network.org/) aggregates these guidelines to improve discoverability^58^. There are also guidelines for improving the reporting of research planning; for example, the Preferred Reporting Items for Systematic Reviews and Meta-analyses (PRISMA) statement for reporting of systematic reviews and meta-analyses^59^, and PRISMA-P for protocols of systematic reviews^60^. The Preregistration Challenge workflow and the pre-registration recipe for social-behavioural research^61^ also illustrate guidelines for reporting research plans.

The success of reporting guidelines depends on their adoption and effective use. The social and behavioural sciences are behind the biomedical sciences in their adoption of reporting guidelines for research, although with rapid adoption of the TOP guidelines and related developments by journals and funders that gap may be closing. However, improved reporting may be insufficient on its own to maximize research quality. Reporting guidelines are easily perceived by researchers as bureaucratic exercises rather than means of improving research and reporting. Even with pre-registration of clinical trials, one study observed that just 13% of trials published outcomes completely consistent with the pre-registered commitments. Most publications of the trials did not report pre-registered outcomes and added new outcomes that were not part of the registered design (see www.COMPare-trials.org). Franco and colleagues observed similar findings in psychology^62^; using protocol pre-registrations and public data from the Time-sharing Experiments for the Social Sciences project (http://www.tessexperiments.org/), they found that 40% of published reports failed to mention one or more of the experimental conditions of the experiments, and approximately 70% of published reports failed to mention one or more of the outcome measures included in the study. Moreover, outcome measures that were not included were much more likely to be negative results and associated with smaller effect sizes than outcome measures that were included.

The positive outcome of reporting guidelines is that they make it possible to detect and study these behaviours and their impact. Otherwise, these behaviours are simply unknowable in any systematic way. The negative outcome is the empirical evidence that reporting guidelines may be necessary, but will not alone be sufficient, to address reporting biases. The impact of guidelines and how best to optimize their use and impact will be best assessed by randomized trials (see Box 4).

## Reproducibility

In this section we describe measures that can be implemented to support verification of research (including, for example, sharing data and methods).

### Promoting transparency and open science

Science is a social enterprise: independent and collaborative groups work to accumulate knowledge as a public good. The credibility of scientific claims is rooted in the evidence supporting them, which includes the methodology applied, the data acquired, and the process of methodology implementation, data analysis and outcome interpretation. Claims become credible by the community reviewing, critiquing, extending and reproducing the supporting evidence. However, without transparency, claims only achieve credibility based on trust in the confidence or authority of the originator. Transparency is superior to trust.

Open science refers to the process of making the content and process of producing evidence and claims transparent and accessible to others. Transparency is a scientific ideal, and adding ‘open’ should therefore be redundant. In reality, science often lacks openness: many published articles are not available to people without a personal or institutional subscription, and most data, materials and code supporting research outcomes are not made accessible, for example, in a public repository (refs 63,64; Box 5).

Very little of the research process (for example, study protocols, analysis workflows, peer review) is accessible because, historically, there have been few opportunities to make it accessible even if one wanted to do so. This has motivated calls for open access, open data and open workflows (including analysis pipelines), but there are substantial barriers to meeting these ideals, including vested financial interests (particularly in scholarly publishing) and few incentives for researchers to pursue open practices. For example, current incentive structures promote the publication of ‘clean’ narratives, which may require the incomplete reporting of study procedures or results. Nevertheless, change is occurring. The TOP guidelines^54,65^ promote open practices, while an increasing number of journals and funders require open practices (for example, open data), with some offering their researchers free, immediate open-access publication with transparent post-publication peer review (for example, the Wellcome Trust, with the launch of Wellcome Open Research). Policies to promote open science can include reporting guidelines or specific disclosure statements (see Box 6). At the same time, commercial and non-profit organizations are building new infrastructure such as the Open Science Framework to make transparency easy and desirable for researchers.

## Evaluation

In this section we describe measures that can be implemented when evaluating research (including, for example, peer review).

### Diversifying peer review

For most of the history of scientific publishing, two functions have been confounded — evaluation and dissemination. Journals have provided dissemination via sorting and delivering content to the research community, and gatekeeping via peer review to determine what is worth disseminating. However, with the advent of the internet, individual researchers are no longer dependent on publishers to bind, print and mail their research to subscribers. Dissemination is now easy and can be controlled by researchers themselves. For example, preprint services (arXiv for some physical sciences, bioRxiv and PeerJ for the life sciences, engrXiv for engineering, PsyArXiv for psychology, and SocArXiv and the Social Science Research Network (SSRN) for the social sciences) facilitate easy sharing, sorting and discovery of research prior to publication. This dramatically accelerates the dissemination of information to the research community.

With increasing ease of dissemination, the role of publishers as a gatekeeper is declining. Nevertheless, the other role of publishing — evaluation — remains a vital part of the research enterprise. Conventionally, a journal editor will select a limited number of reviewers to assess the suitability of a submission for a particular journal. However, more diverse evaluation processes are now emerging, allowing the collective wisdom of the scientific community to be harnessed^66^. For example, some preprint services support public comments on manuscripts, a form of pre-publication review that can be used to improve the manuscript. Other services, such as PubMed Commons and PubPeer, offer public platforms to comment on published works facilitating post-publication peer review. At the same time, some journals are trialling ‘results-free’ review, where editorial decisions to accept are based solely on review of the rationale and study methods alone (that is, results-blind)^67^.

Both pre- and post-publication peer review mechanisms dramatically accelerate and expand the evaluation process^68^. By sharing preprints, researchers can obtain rapid feedback on their work from a diverse community, rather than waiting several months for a few reviews in the conventional, closed peer review process. Using post-publication services, reviewers can make positive and critical commentary on articles instantly, rather than relying on the laborious, uncertain and lengthy process of authoring a commentary and submitting it to the publishing journal for possible publication, eventually.

As public forms of pre- and post-publication review, these new services introduce the potential for new forms of credit and reputation enhancement^69^. In the conventional model, peer review is done privately, anonymously and purely as a service. With public commenting systems, a reviewer that chooses to be identifiable may gain (or lose) reputation based on the quality of review. There are a number of possible and perceived risks of non-anonymous reviewing that reviewers must consider and research must evaluate, but there is evidence that open peer review improves the quality of reviews received^70^. The opportunity for accelerated scholarly communication may both improve the pace of discovery and diversify the means of being an active contributor to scientific discourse.

## Incentives

Publication is the currency of academic science and increases the likelihood of employment, funding, promotion and tenure. However, not all research is equally publishable. Positive, novel and clean results are more likely to be published than negative results, replications and results with loose ends; as a consequence, researchers are incentivized to produce the former, even at the cost of accuracy^40^. These incentives ultimately increase the likelihood of false positives in the published literature^71^. Shifting the incentives therefore offers an opportunity to increase the credibility and reproducibility of published results. For example, with simulations, Munafò and Higginson developed an optimality model that predicted the most rational research strategy, in terms of the proportion of research effort spent on seeking novel results rather than on confirmatory studies, and the amount of research effort per exploratory study^72^. This showed that, for parameter values derived from the scientific literature, researchers acting to maximize their ‘fitness’ should spend most of their effort seeking novel results and conduct small studies that have a statistical power of only 10-40%. Critically, their model suggests that altering incentive structures, by considering more of a researcher’s output and giving less weight to strikingly novel findings when making appointment and promotion decisions, would encourage a change in researcher behaviour that would ultimately improve the scientific value of research.

**Figure 2:**
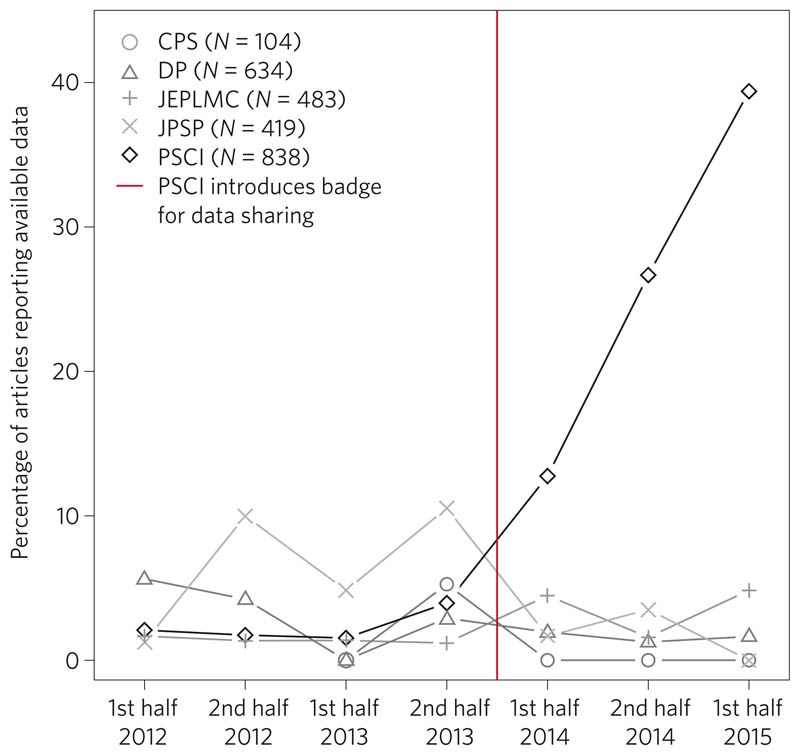
The impact of introducing badges for data sharing. In January 2014, the journal *Psychological Science* (PSCI) introduced badges for articles with open data. Immediately afterwards, the proportion of articles with open data increased steeply, and by October 2015, 38% of articles in *Psychological Science* had open data. For comparison journals *(Clinical Psychological Science* (CPS), *Developmental Psychology* (DP), *Journal of Experimental Psychology: Learning, Memory and Cognition* (JEPLMC) and *Journal of Personality and Social Psychology* (JPSP)) the proportion of articles with open data remained uniformly low. Figure adapted from ref. 75, PLoS.

Funders, publishers, societies, institutions, editors, reviewers and authors all contribute to the cultural norms that create and sustain dysfunctional incentives. Changing the incentives is therefore a problem that requires a coordinated effort by all stakeholders to alter reward structures. There will always be incentives for innovative outcomes — those who discover new things will be rewarded more than those who do not. However, there can also be incentives for efficiency and effectiveness — those who conduct rigorous, transparent and reproducible research could be rewarded more than those who do not. There are promising examples of effective interventions for nudging incentives. For example, journals are adopting badges to acknowledge open practices (Fig. 2), Registered Reports as a results-blind publishing model (see Box 3) and TOP guidelines to promote openness and transparency. Funders are also adopting transparency requirements, and piloting funding mechanisms to promote reproducibility such as the Netherlands Organisation for Scientific Research (NWO) and the US National Science Foundation’s Directorate of Social, Behavioral and Economic Sciences, both of which have announced funding opportunities for replication studies. Institutions are wrestling with policy and infrastructure adjustments to promote data sharing, and there are hints of open-science practices becoming part of hiring and performance evaluation (for example, http://www.nicebread.de/open-science-hiring-practices/). Collectively, and at scale, such efforts can shift incentives such that what is good for the scientist is also good for science — rigorous, transparent and reproducible research practices producing credible results.

## Conclusion

The challenges to reproducible science are systemic and cultural, but that does not mean they cannot be met. The measures we have described constitute practical and achievable steps toward improving rigor and reproducibility. All of them have shown some effectiveness, and are well suited to wider adoption, evaluation and improvement. Equally, these proposals are not an exhaustive list; there are many other nascent and maturing ideas for making research practices more efficient and reliable^73^. Offering a solution to a problem does not guarantee its effectiveness, and making changes to cultural norms and incentives can spur additional behavioural changes that are difficult to anticipate. Some solutions may be ineffective or even harmful to the efficiency and reliability of science, even if conceptually they appear sensible.

The field of metascience (or metaresearch) is growing rapidly, with over 2,000 relevant publications accruing annually^16^. Much of that literature constitutes the evaluation of existing practices and the identification of alternative approaches. What was previously taken for granted may be questioned, such as widely used statistical methods; for example, the most popular methods and software for spatial extent analysis in fMRI imaging were recently shown to produce unacceptably high false-positive rates^74^. Proposed solutions may also give rise to other challenges; for example, while replication is a hallmark for reinforcing trust in scientific results, there is uncertainty about which studies deserve to be replicated and what would be the most efficient replication strategies. Moreover, a recent simulation suggests that replication alone may not suffice to rid us of false results^71^.

These cautions are not a rationale for inaction. Reproducible research practices are at the heart of sound research and integral to the scientific method. How best to achieve rigorous and efficient knowledge accumulation is a scientific question; the most effective solutions will be identified by a combination of brilliant hypothesizing and blind luck, by iterative examination of the effectiveness of each change, and by a winnowing of many possibilities to the broadly enacted few. True understanding of how best to structure and incentivize science will emerge slowly and will never be finished. That is how science works. The key to fostering a robust metascience that evaluates and improves practices is that the stakeholders of science must not embrace the status quo, but instead pursue self-examination continuously for improvement and self-correction of the scientific process itself.

As Richard Feynman said, “The first principle is that you must not fool yourself - and you are the easiest person to fool.”

## Figures and Tables

**Figure 1:**
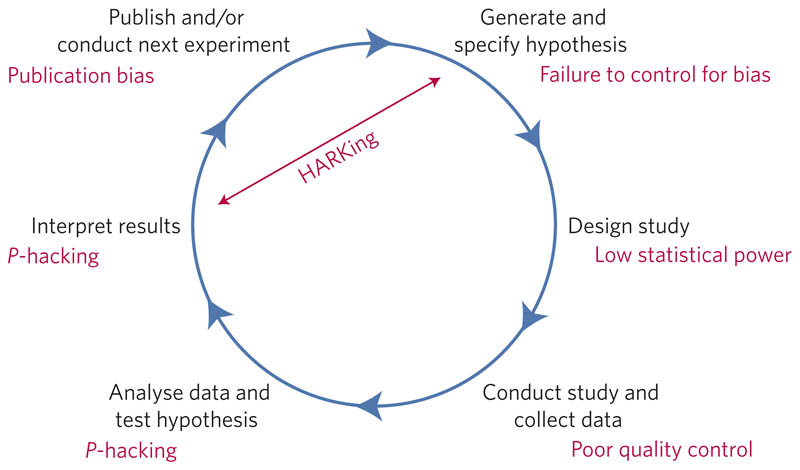
Threats to reproducible science. An idealized version of the hypothetico-deductive model of the scientific method is shown. Various potential threats to this model exist (indicated in red), including lack of replication^5^, hypothesizing after the results are known (HARKing)^7^, poor study design, low statistical power^2^, analytical flexibility^51^, *P*-hacking^4^, publication bias^3^ and lack of data sharing^6^. Together these will serve to undermine the robustness of published research, and may also impact on the ability of science to self-correct.

**Table 1:** A manifesto for reproducible science.

Theme	Proposal	Examples of initiatives/potential solutions (extent of current adoption)	Stakeholder(s)
Methods	Protecting against cognitive biases	All of the initiatives listed below (* to ****) Blinding (**)	J, F
	Improving methodological training	Rigorous training in statistics and research methods for future researchers (*) Rigorous continuing education in statistics and methods for researchers (*)	I, F
	Independent methodological support	Involvement of methodologists in research (**) Independent oversight (*)	F
	Collaboration and team science	Multi-site studies/distributed data collection (*) Team-science consortia (*)	I, F
Reporting and dissemination	Promoting study pre-registration	Registered Reports (*) Open Science Framework (*)	J, F
	Improving the quality of reporting	Use of reporting checklists (**) Protocol checklists (*)	J
	Protecting against conflicts of interest	Disclosure of conflicts of interest (***) Exclusion/containment of financial and non-financial conflicts of interest (*)	J
Reproducibility	Encouraging transparency and open science	Open data, materials, software and so on (* to **) Pre-registration (**** for clinical trials, * for other studies)	J, F, R
Evaluation	Diversifying peer review	Preprints (* in biomedical/behavioural sciences, **** in physical sciences) Pre- and post-publication peer review, for example, Publons, PubMed Commons (*)	J
Incentives	Rewarding open and reproducible practices	Badges (*) Registered Reports (*) Transparency and Openness Promotion guidelines (*) Funding replication studies (*) Open science practices in hiring and promotion (*)	J, I, F

Estimated extent of current adoption: *, <5%; **, 5–30%; ***, 30–60%; ****, >60%. Abbreviations for key stakeholders: J, journals/publishers; F, funders; I, institutions; R, regulators.

## Box 1 Independent oversight: the case of CHDI Foundation.

CHDI Foundation — a privately-funded non-profit drug-development organization targeting Huntington’s disease — convened a working group in 2013 to identify practical and viable steps that could be taken to help ensure the rigor of their research^76^. One concrete product of this meeting was the establishment of the Independent Statistical Standing Committee (ISSC; http://chdi-foundation.org/independent-statistical-standing-committee/) designed to provide independent, unbiased and objective evaluation and expert advice regarding all aspects of experimental design and statistics. CHDI has made this resource available to the wider Huntington’s disease research community on a priority basis. The ISSC is comprised of individuals with specific expertise in research design and statistics. Critically, committee members are not themselves engaged in Huntington’s disease research, and have no investment in study results, or other conflicts of interest. The committee provides a number of services, including (but not limited to) provision of expert assistance in developing protocols and statistical analysis plans, and evaluation of prepared study protocols. Their oversight and input, particularly at the study design stage, may mitigate low statistical power, inadequate study design, and flexibility in data analysis and reporting^6,71^. As recently highlighted, “asking questions at the design stage can save headaches at the analysis stage: careful data collection can greatly simplify analysis and make it more rigorous”^77^.

## Box 2 Distributed student projects.

Student assessment requirements, and limited access to populations of interest, may hinder extensive collaboration within a single institution, but it could be achieved across multiple institutions in the form of a distributed student project. Under this model, academics and students from several institutions would form a consortium, collaboratively develop a research question, protocol and analysis plan, and publicly pre-register it prior to data collection. The protocol would be implemented by each student at each participating centre, and the resulting data pooled for analysis. Consortium meetings before and after data collection could be used to integrate training in research design, while offering opportunities for creative input from the students. Conclusions based on results would be mutually agreed in preparation for wider dissemination, using inclusive authorship conventions such as those adopted by genetic consortia. Students would learn rigorous research methods through active participation in research that is sufficiently well designed and conducted to be genuinely meaningful. Critically, collaborative team science would be instilled at an early stage of training.

The Collaborative Replications and Education Project (CREP; https://osf.io/wfc6u/) is an example of this concept in psychology, albeit in a more centralized form. A coordinating team identifies recently published research that could be replicated in the context of a semester-long undergraduate course on research methods. A central commons provides the materials and guidance to incorporate the replications into projects or classes, and the data collected across sites are aggregated into manuscripts for publication. The Pipeline^78^ and Many Labs^44,45^ projects also offer opportunities to contribute to large-scale replication efforts with coordinated data collection across many locations simultaneously.

## Box 3 Registered Reports.

The Registered Reports (RR) initiative seeks to eliminate various forms of bias in hypothesis-driven research^52,53^, and in particular, the evaluation of a study based on the results. Unlike conventional journal articles, RRs split the peer review process into two stages, before and after results are known. At the first stage, reviewers and editors assess a detailed protocol that includes the study rationale, procedure and a detailed analysis plan. Following favourable reviews (and probably revision to meet strict methodological standards), the journal offers in-principle acceptance: publication of study outcomes is guaranteed provided the authors adhere to the approved protocol, the study meets pre-specified quality checks, and conclusions are appropriately evidence-bound. Once the study is completed, the authors resubmit a complete manuscript that includes the results and discussion. The article is published at the end of this two-stage process. By accepting articles before results are known, RRs prevent publication bias. By reviewing the hypotheses and analysis plans in advance, RRs should also help neutralize *P-* hacking and HARKing (hypothesizing after the results are known) by authors, and CARKing (critiquing after the results are known) by reviewers with their own investments in the research outcomes, although empirical evidence will be required to confirm that this is the case.

Perhaps the most commonly voiced objection to RRs is that the format somehow limits exploration or creativity by requiring authors to adhere to a pre-specified methodology. However, RRs place no restrictions on creative analysis practices or serendipity. Authors are free to report the outcomes of any unregistered exploratory analyses, provided such tests are clearly labelled as *post hoc.* Thus, the sole requirement is that exploratory outcomes are identified transparently as exploratory (for a list of frequently asked questions see https://cos.io/rr/#faq). Of course, RRs are not intended for research that is solely exploratory.

As of November 2016, RRs have been adopted by over 40 journals, including *Nature Human Behaviour,* covering a wide range of life, social and physical sciences (for a curated list see https://cos.io/rr/#journals). The concept also opens the door to alternative forms of research funding that place a premium on transparency and reproducibility. For example, authors could submit a detailed proposal before they have funding for their research. Following simultaneous review by both the funder and the journal, the strongest proposals would be offered financial support by the funder and in-principle acceptance for publication by the journal (https://cos.io/rr/#funders).

## Box 4 Evidence for the effectiveness of reporting guidelines.

In medicine there is strong evidence for the effectiveness of CONSORT guidelines — journals that do not endorse the CONSORT statement show poorer reporting quality compared with endorsing journals^79^. For the ARRIVE (Animal Research: Reporting of *In Vivo* Experiments) guidelines^80^, studies comparing the reporting of ARRIVE items in specific fields of research before and after the guidelines were published report mixed results^81–83^. A randomized controlled trial is in progress to assess the impact of mandating a completed ARRIVE checklist with manuscript submissions on the quality of reporting in published articles (https://ecrf1.clinicaltrials.ed.ac.uk/iicarus). The success of these efforts will require journals and funders to adopt guidelines and support the community’s iterative evaluation and improvement cycle.

## Box 5 Data sharing.

Sharing data in public repositories offers field-wide advantages in terms of accountability, data longevity, efficiency and quality (for example, reanalyses may detect crucial mistakes or even data fabrication)^84^. Unfortunately, many scientific disciplines, including most of those devoted to the study of human behaviour, do not have a culture that values open data^6^. In the past, data sharing has rarely been enforced or facilitated. Recent initiatives, however, aim to change the normative culture. Hopefully, these initiatives will change the culture on data sharing. Once accepted as the norm, we doubt that data sharing will ever go out of fashion.

### Transparency and Openness Promotion (TOP)

In 2015, Nosek and colleagues^54^ proposed author guidelines to help journals and funders adopt transparency and reproducibility policies. As of November 2016 there were 757 journal and 64 organization signatories to the TOP guidelines. For example, the journal *Science* decided to “publish papers only if the data used in the analysis are available to any researcher for purposes of reproducing or extending the analysis”^65^ and the conglomerate of Springer Nature journals adopted similar data-sharing policies.

### The Peer Reviewers’ Openness Initiative

Researchers who sign this initiative (https://opennessinitiative.org) pledge that as reviewers they will not offer comprehensive review for any manuscript that does not make data publicly available without a clear reason^85^.
